# An unusual presentation of girl with down syndrome: Van-Wyk Grumbach syndrome

**DOI:** 10.1186/1687-9856-2013-S1-P203

**Published:** 2013-10-03

**Authors:** Han Hyuk Lim, Hong Ryang Kil, Jae Young Kim

**Affiliations:** 1 Department Of Pediatrics, Chungnam National University Hospital

## 

Van Wyk-Grumbach syndrome is a rare disease characterized by precocious puberty associated with prolonged hypothyroidism and multicystic enlarged ovaries.A9 year-old girl with Down syndrome visited our hospital for early menarche. At birth, she showed subclinical hypothyroidism (11.8 mg/dL of thyroxine (T4) and 6.05 mIU/mL of thyroid stimulating hormone (TSH)), but she had not been followed up in our clinic. On physical examination, pubertal Tanner stage was breast II and pubic hair I. Laboratory findings were as follows; 0.30 ng/dL of free T4, 81.30 uIU/mL of TSH, 0.1 IU/L of luteinizing hormone, and 6.35 IU/L of follicle-stimulating hormone. Her bone age was 6 years. Her pelvic sonogram revealed multiple cysts in both enlarged ovaries. She was diagnosed with Van Wyk-Grumbach syndrome. Levothyroxine treatment at a dose of 0.05mg/day was started. Regression of breast development was obtained after 2 months and her vaginal bleeding did not recur.

